# Sulfonate-Modified Polystyrene Nanoparticle at Precited Environmental Concentrations Induces Transgenerational Toxicity Associated with Increase in Germline Notch Signal of *Caenorhabditis elegans*

**DOI:** 10.3390/toxics11060511

**Published:** 2023-06-06

**Authors:** Wenmiao He, Aihua Gu, Dayong Wang

**Affiliations:** 1 Key Laboratory of Environmental Medicine Engineering of Ministry of Education, Medical School, Southeast University, Nanjing 210009, China; 2 School of Public Health, Nanjing Medical University, Nanjing 211166, China; 3 Shenzhen Ruipuxun Academy for Stem Cell & Regenerative Medicine, Shenzhen 518122, China

**Keywords:** *C. elegans*, transgenerational toxicity, nanoplastic, Notch signal

## Abstract

Recently, the transgenerational toxicity of nanoplastics has received increasing attention. *Caenorhabditis elegans* is a useful model to assess the transgenerational toxicity of different pollutants. In nematodes, the possibility of early-life exposure to sulfonate-modified polystyrene nanoparticle (PS-S NP) causing transgenerational toxicity and its underlying mechanisms were investigated. After exposure at the L1-larval stage, transgenerational inhibition in both locomotion behavior (body bend and head thrash) and reproductive capacity (number of offspring and fertilized egg number in uterus) was induced by 1–100 μg/L PS-S NP. Meanwhile, after exposure to 1–100 μg/L PS-S NP, the expression of germline *lag-2* encoding Notch ligand was increased not only at the parental generation (P0-G) but also in the offspring, and the transgenerational toxicity was inhibited by the germline RNA interference (RNAi) of *lag-2*. During the transgenerational toxicity formation, the parental LAG-2 activated the corresponding Notch receptor GLP-1 in the offspring, and transgenerational toxicity was also suppressed by *glp-1* RNAi. GLP-1 functioned in the germline and the neurons to mediate the PS-S NP toxicity. In PS-S NP-exposed nematodes, germline GLP-1 activated the insulin peptides of INS-39, INS-3, and DAF-28, and neuronal GLP-1 inhibited the DAF-7, DBL-1, and GLB-10. Therefore, the exposure risk in inducing transgenerational toxicity through PS-S NP was suggested, and this transgenerational toxicity was mediated by the activation of germline Notch signal in organisms.

## 1. Introduction

It has been reported that a total of more than 8 billion tons of plastics have been synthesized and produced since the 1950s [1]. One of the important causes of plastic pollution is the formation of plastic debris with small sizes, including microplastics and nanoplastics, as categorized by their sizes [2,3]. Considering the environmental burden of waste plastics, a large amount of microplastics and nanoplastics are likely to be formed in the environment [4]. Due to their smaller size, the distribution and potential environmental risks of nanoplastics have received increasing attention [5,6]. Nanoplastics can be found in several ecosystems, such as the soil ecosystem [7,8]. Moreover, the further frequent detection of nanoplastics in drinking water, food, and air has suggested the constant exposure of humans to these nanoparticles [9,10].

The exposure routes of nanoplastics for environmental animals and humans may mainly contain oral, dermal, and inhalation routes [11,12]. More importantly, once bioavailable, nanoplastics can further cross primary biological barriers to reach other tissues and system circulation [13,14]. After ingestion, the excretory system of environmental animals may expel more than 90% of nanoplastics in feces [15], which implies that the excretory system is the main targeted organ at the parental generation (P0-G). Nanoplastic exposure induces toxicity on environmental animals (such as zebrafish and Daphnia) and human health [16,17,18]. Several factors have been suggested to likely affect the toxicity induction of nanoplastics, including size, charge, dose, and leachables [19]. Moreover, the other adsorbed pollutants may also imply the interaction between nanoplastics and pollutants, such as the enhancement in contaminant transport [20,21]. Recently, the transgenerational or multigenerational toxicities of nanoplastics were further detected in Daphnia and mice [22,23,24].

The *Caenorhabditis elegans* has been used for the toxicological study of various pollutants [25,26,27,28,29]. At P0-G, toxicity in the development and function of the nervous system could be induced by exposure to polystyrene nanoparticles (PS NPs) [30,31]. PS NP exposure caused reproductive reduction and altered biochemical metabolisms [32,33]. Largely due to its short lifecycle and lifespan [34], *C. elegans* provide a useful platform for the transgenerational toxicity assessment of pollutants [35,36]. The toxic effects of PS NP on locomotion and reproduction could be further observed in the offspring of exposed nematodes [37,38]. During the control of transgenerational PS NP toxicity, some epigenetic regulation signals (such as histone methyltransferases (MET-2 and SET-6) and microRNAs (*mir-38*)) have been proven to have important functions [39,40,41].

The sulfonate-modified polystyrene nanoparticle (PS-S NP) is a form of -SO_3_H-modified PS NP. The -SO_3_H-modified polystyrene can be used in the chelating agent, extracting metals by adsorption, and the biosensor [42,43,44]. PS-S NP caused more severe damage on the development of dopaminergic neurons and locomotion than pristine PS NP [45]. However, the possible effect of PS-S NP in inducing transgenerational toxicity and the underlying mechanism are largely unclear. In *C. elegans*, locomotion and reproduction are two endpoints more frequently applied for evaluating the transgenerational toxicity of pollutants [46,47]. Using *C. elegans* as the animal model, we first used locomotion and reproduction as endpoints to evaluate the toxicity of S-PS-NP in causing transgenerational toxicity. In organisms, the Notch signaling pathway mediates important cell–cell interactions [48,49]. In nematodes, the Notch ligands contain ARG-1, APX-1, LAG-2, and DSL-1-7 [50]. The corresponding Notch receptors are LIN-12 and GLP-1 [51]. Notch receptor GLP-1 is involved in regulating the toxicity of CdTe quantum dots and simulated microgravity [52,53]. Moreover, we focused on the Notch signal in the germline to determine its possible involvement in mediating transgenerational PS-S NP toxicity. Our results highlight the risk of PS-S NP in inducing transgenerational toxicity. In addition, both the germline Notch ligand of LAG-2 and its receptor GLP-1 could act as useful bioindicators for evaluating the transgenerational nanoplastic toxicity.

## 2. Materials and Methods

### 2.1. PS-S NP Characterization

**Figure 1:**
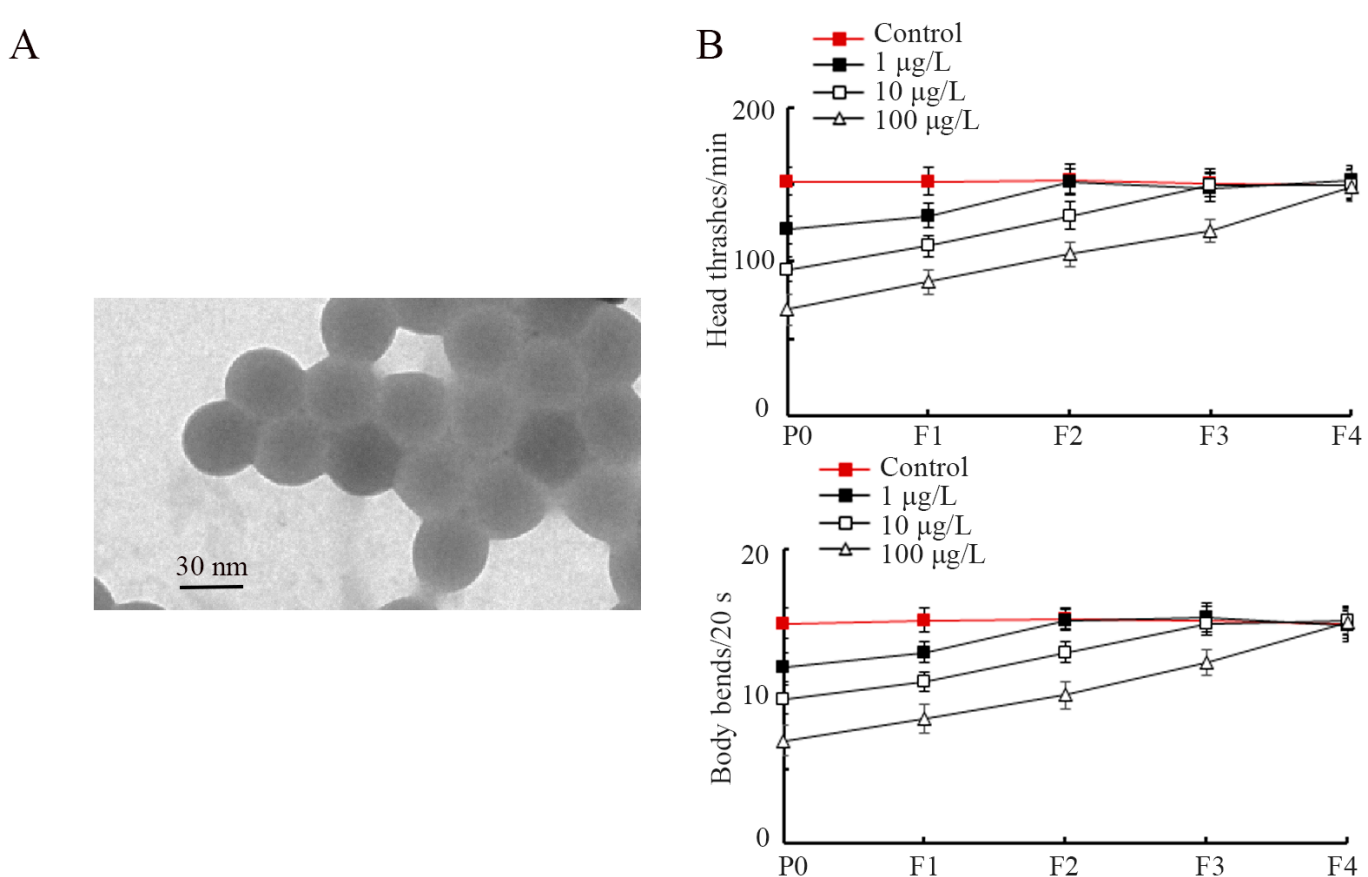
PS-S NP exposure induced transgenerational toxicity on locomotion behavior in nematodes. (**A**) TEM image of PS-S NP before the sonication. (**B**) Effect of PS-S NP exposure on locomotion behaviors of head thrash and body bend transgenerationally. The curves of PS-S NP (1 μg/L), PS-S NP (10 μg/L), and PS-S NP (100 μg/L) showed a significant difference (*p* < 0.01) compared with control.

PS-S NP (35 nm in diameter) was gifted from Dr. Xianzheng Yuan’s lab (Shandong University). PS-S NPs were diluted in K buffer. The PS-S NP morphology was observed under transmission electron microscopy (TEM). As shown in Figure 1A, PS-S NPs were spherical. The dynamic light scattering (DLS) analysis indicated that the PS-S NP was 32.4 ± 3.8 nm in diameter, and no obvious aggregation was observed for PS-S NP suspensions at examined concentrations for at least for three days after sonication. The zeta potential of PS-S NPs was −52.17 ± 4.43 mV. Raman spectrum and Fourier transform infrared (FTIR) spectrum of PS-S NP were described previously [45].

### 2.2. C. elegans Maintenance

*C. elegans* strain information was shown in Table S1. Worms were maintained on nematode growth medium (NGM) seeded with *Escherichia coli* OP50 as the food source [54].

Worm synchronization can be performed when the adult uterus is full of germlines. Firstly, gravid adult worms were collected into 1.5 mL tubes and washed three times to remove OP50. Subsequently, worms were lysed with bleaching solution (2% HOCl, 0.45 M NaOH) [55], and then centrifuged at 3000 rpm for 3 min to collect the released eggs after washing by K buffer. The eggs were incubated in K buffer and hatched into L1-larvae overnight at 20 °C. Finally, the synchronized worms were transferred onto new NGMs with OP50 for corresponding exposures.

### 2.3. Exposure

The synchronized L1-larvae worms were exposed to 1, 10, and 100 μg/L PS-S NPs together with OP50 (4 × 10^6^ CFUs) until adult day-1 (approximately 4.5-day). Meanwhile, PS-S NP suspensions were updated daily. The PS-S NP exposure only performed in P0-G, and the offspring were maintained on normal NGM plates. PS-S NP suspension was sonicated at 40 kHz for 30 min before exposure.

### 2.4. Locomotion Behaviors

Locomotion behavior was assayed by head thrash and body bend [56]. A head thrash is defined as one swing of the nematode body, and a body bend refers to the crawling of one wavelength [57]. After the exposure, at least 40 nematodes from each concentration were placed on new NGM agar without food. After a minute of recovery, the frequency was counted within 1 min (head thrashes) or 20 s (body bends) under a dissection microscope.

### 2.5. Reproductive Capacity

Reproductive capacity was assessed by the endpoints of brood size and number of fertilized eggs in the uterus [58]. At the end of the exposure period, thirty nematodes per treatment were transferred on NGM plates with bacteria, respectively. Brood size was considered as number of offspring until nematodes end up laying eggs [59]. Fertilized egg number in the uterus was counted using the stereomicroscope. The number of hatched eggs was calculated by the percentage of hatched eggs to the total eggs.

### 2.6. Transcriptional Expression Analysis

The total RNA of *C. elegans* was collected by Trizol (Sigma-Aldrich, Milwaukee, Germany) according to the instructions, and the concentration and purity of collected RNA were determined by NanoDrop One (Thermo Scientific, Waltham, MA, USA). The complementary DNA (cDNA) was obtained by M-MuLV Reverse Transcriptase (Sangon Biotech, Shanghai, China), and cDNA in each group was reversed from RNA at the same amount (500 ng). The polymerase chain reaction (qRT-PCR) was performed with SYBR Premix Ex Taq (Takara, Kyoto, Japan) in StepOnePlus real-time PCR system (Applied Biosystems, Waltham, MA, USA). The transcriptional expressions of target genes were calculated by Δ cycle threshold (ΔCt) method, and *tba-1* acted as the reference gene [60]. To analyze the gene expression in germline, the intact gonads were isolated. Three replicates were performed. The primer sequences were provided in Table S2. RNA and cDNA were stored at −80 °C and −20 °C, respectively.

### 2.7. RNA Interference (RNAi)

The double-stranded RNAs (dsRNA) of target genes were cloned into plasmid L4440 after double enzyme digestion, and then the recombinant plasmid was transferred into *E. coli* HT115 [61]. The transferred HT115 was screened in Luria-Bertani (LB) agar with ampicillin and tetracycline. Subsequently, the plasmid of screened HT115 was exacted for sequence verification. The HT115 containing dsRNA was amplified overnight and incubated with 0.4 mM IPTG for 4 h, then seeded on NGMs. As for RNAi assay, *C. elegans* were fed with *E. coli* HT115 containing specific dsRNA, and HT115 expressing L4440 only acted as the control [62]. The efficiency for RNAi of genes is shown in Figures S1 and S2.

### 2.8. Data Analysis

SPSS v19.0 software was applied for the statistical tests. One-way analysis of variance (ANOVA), two-way ANOVA, and Student’s *t*-test were applied for examining significance between groups. The curves of the transgenerational assay were analyzed by Kaplan–Meier analysis, followed by the log-rank test. *p <* 0.01 (**) was considered statistically significant.

## 3. Results

### 3.1. Exposure to PS-S NP Induced Transgenerational Locomotion Inhibition

Firstly, we assessed the effects of early-life exposure to PS-S NP on transgenerational alteration in locomotion behavior. Parental exposure to 1, 10, and 100 μg/L PS-S NP significantly inhibited both head thrash and body bend transgenerationally, and this transgenerational inhibition exhibited a concentration-dependent manner (Figure 1B). The transgenerational inhibition in locomotion behavior by 1, 10, and 100 μg/L PS-S NP was recovered at F2-G, F3-G, and F4-G, respectively (Figure 1B). These results showed that the parental exposure to PS-S NP caused the transgenerational locomotion disorder in *C. elegans*.

### 3.2. Exposure to PS-S NP-Induced Transgenerational Inhibition in Reproductive Capacity

**Figure 2:**
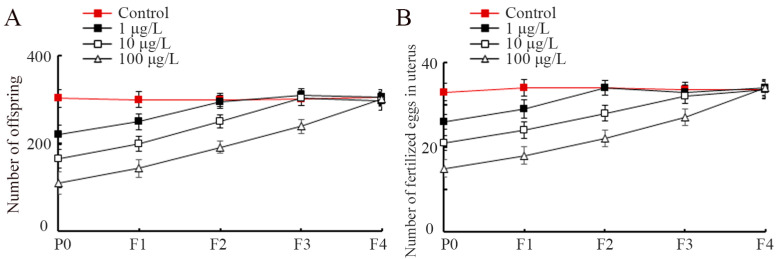
PS-S NP exposure-induced transgenerational toxicity on reproductive capacity in the nematodes. (**A**) Effect of PS-S NP exposure on brood size transgenerationally. (**B**) Effect of S-PS-NP exposure on number of fertilized eggs in the uterus transgenerationally. The curves of PS-S NP (1 μg/L), PS-S NP (10 μg/L), and PS-S NP (100 μg/L) showed a significant difference (*p* < 0.01) compared with control.

Next, we evaluated the effects of early-life exposure to PS-S NP on transgenerational alterations in reproductive capacity. Similar to the PS-S NP-induced transgenerational locomotion inhibition, parental exposure to 1–100 μg/L PS-S NP significantly inhibited reproductive capacity, and this transgenerational inhibition also had concentration-dependent property (Figure 2A,B). The transgenerational suppression in brood size and fertilized egg number in the uterus could be also recovered at F2-G, F3-G, and F4-G, respectively (Figure 2A,B). Therefore, PS-S NP exposure further induced the transgenerational reproduction suppression.

### 3.3. Exposure to PS-S NP Induced Transgenerational Increase in Expression of Germline lag-2

**Figure 3:**
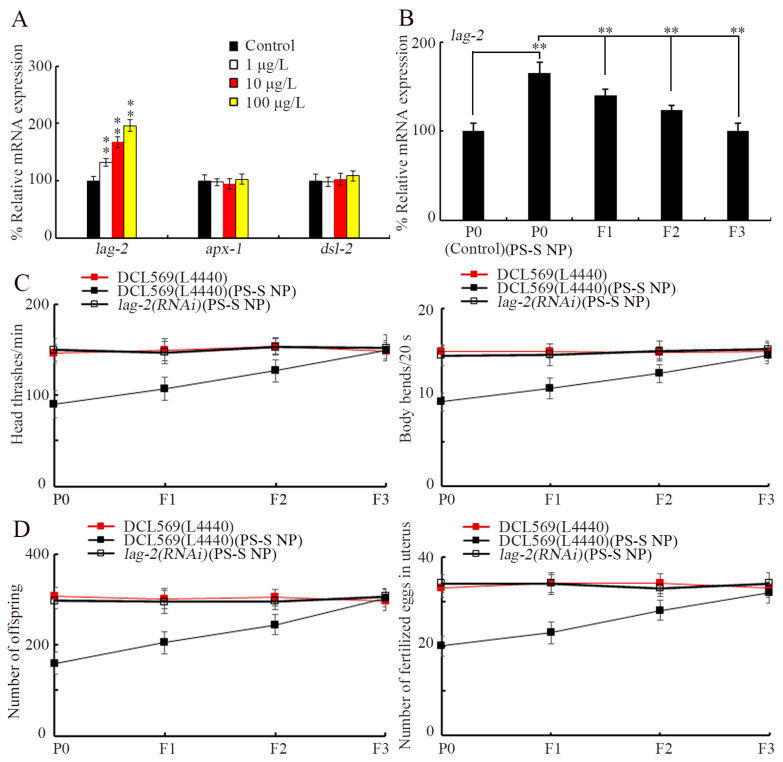
Requirement of germline LAG-2 for controlling the transgenerational PS-S NP toxicity. (**A**) Effect of PS-S NP exposure on expressions of the germlines *lag-2*, *apx-1*, and *dsl-2* at P0-G. ** *p* < 0.01 vs. control. (**B**) Germline *lag-2* expressions from P0-G to F3-G after parental exposure to 10 μg/L PS-S NP. ** *p* < 0.01. (**C**) Effect of the germline *lag-2* RNAi on transgenerational toxicity of 10 μg/L PS-S NP in inhibiting locomotion. (**D**) Effect of the germline *lag-2* RNAi on transgenerational toxicity of 10 μg/L PS-S NP in reducing reproductive capacity. Curves of DCL569(L4440)(PS-S NP) showed a significant difference (*p* < 0.01) compared with DCL569(L4440). After the PS-S NP exposure, the curves of *lag-2(RNAi)* showed a significant difference (*p* < 0.01) compared with DCL569(L4440).

Among the Notch ligands, LAG-2, APX-1, and DSL-2 can be expressed in the germlines (https://wormbase.org, accessed on 1 January 2023). At P0-G, 1–100 μg/L PS-S NP did not alter the expressions of germline *apx-1* and *dsl-2* (Figure 3A). In contrast, the expression of germline *lag-2* was increased by 1–100 μg/L PS-S NP at P0-G, and this increase in *lag-2* expression was concentration-dependent (Figure 3A). Moreover, after parental exposure to 10 μg/L PS-S NP, a significant increase in germline *lag-2* expression was also detected from F1-G to F2-G, and recovered at F3-G (Figure 3B).

### 3.4. Germline Notch Ligand LAG-2 Was Required for Induction of Transgenerational PS-S NP Toxicity

To further investigate the role of *lag-2* in transgenerational PS-S NP toxicity, germline RNAi of *lag-2* was performed using strain DCL569 [63]. The RNAi of *lag-2* using the bacterial feeding method significantly reversed the alterations in body bend, head thrash, brood size, and fertilized egg number in the uterus in 10 μg/L PS-S NP exposed parents, as well as in their offspring (Figure 3C,D). Namely, germline RNAi of *lag-2* showed the resistance to transgenerational toxicity.

### 3.5. Exposure to PS-S NP Caused Transgenerational Increase in Expression of glp-1

**Figure 4:**
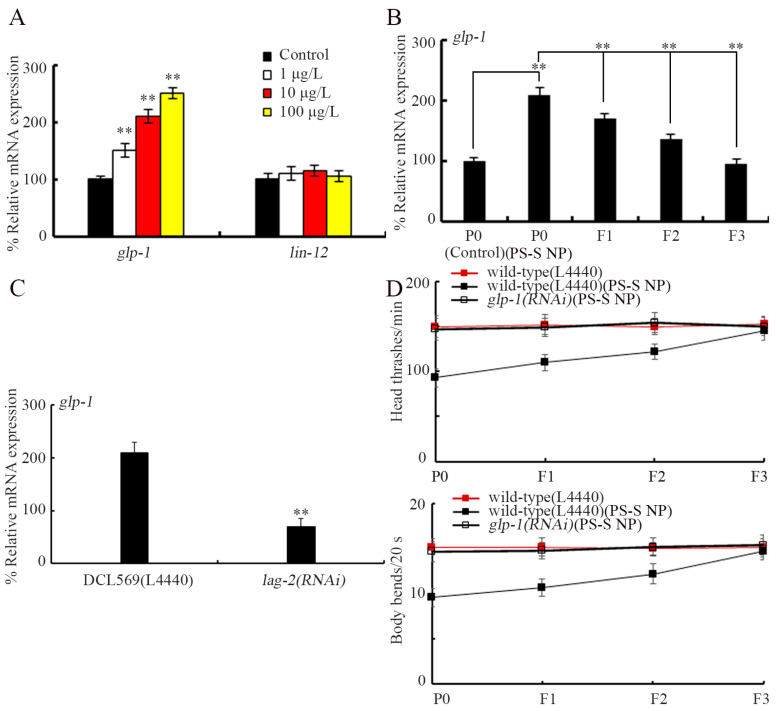
Requirement of Notch receptor GLP-1 for controlling the transgenerational PS-S NP toxicity. (**A**) Effect of PS-S NP exposure on expressions of *glp-1* and *lin-12* at P0-G. ** *p* < 0.01 vs. control. (**B**) *glp-1* expression from P0-G to F3-G after parental exposure to 10 μg/L PS-S NP. ** *p* < 0.01. (**C**) Effect of parental germline RNAi of *lag-2* on *glp-1* expression in the offspring of 10 μg/L PS-S NP exposed nematodes. The RNAi of *lag-2* was performed at P0-G, and the *glp-1* expression was analyzed at F1-G. ** *p* < 0.01. (**D**) Effect of *glp-1* RNAi on transgenerational toxicity of 10 μg/L PS-S NP in inhibiting locomotion. Curves of wild-type (L4440)(PS-S NP) showed a significant difference (*p* < 0.01) compared with wild-type (L4440). After the PS-S NP exposure, the curves of *glp-1(RNAi)* showed a significant difference (*p* < 0.01) compared with wild-type (L4440).

We next examined the effect of PS-S NP on the expressions of genes encoding Notch receptors. Among the examined two Notch receptor genes, 1–100 μg/L PS-S NP did not alter *lin-12* expression, whereas the *glp-1* expression was increased by 1–100 μg/L PS-S NP (Figure 4A). Meanwhile, after parental exposure to 10 μg/L PS-S NP, an increase in *glp-1* expression could also be observed at F1-G and F2-G, and recovered until F3-G (Figure 4B). Moreover, after germline RNAi of *lag-2* in 10 μg/L PS-S NP at P0-G, we further detected a significant decrease in *glp-1* expression at F1-G (Figure 4C).

### 3.6. Notch Receptor GLP-1 Was Involved in Induction of Transgenerational PS-S NP Toxicity

We further carried out RNAi of *glp-1* to investigate its possible function in controlling transgenerational PS-S NP toxicity. Considering *glp-1* mutant nematodes have germline-loss phenotype [64,65], we employed locomotion as the endpoint. The transgenerational inhibition in locomotion observed in 10 μg/L PS-S NP-exposed animals was prevented by RNAi of *glp-1* (Figure 4D). Therefore, both Notch ligand/LAG-2 and Notch receptor/GLP-1 were essential for the formation of transgenerational PS-S NP toxicity.

### 3.7. Tissue-Specific Activities of GLP-1 in Controlling Transgenerational PS-S NP Toxicity

**Figure 5:**
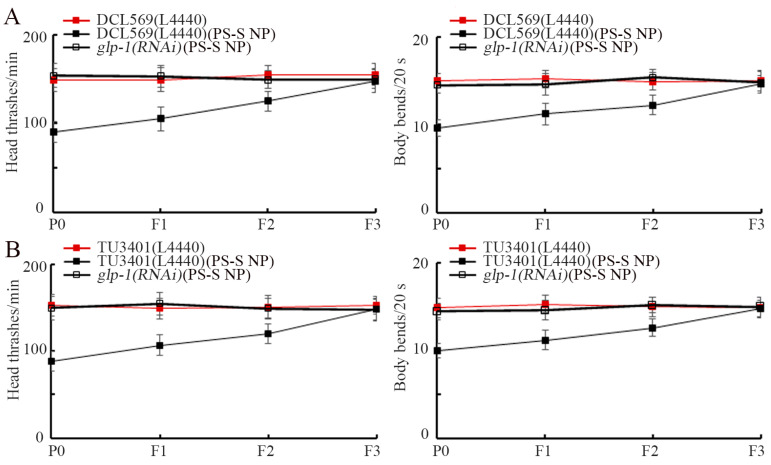
Tissue-specific activity of GLP-1 in controlling the transgenerational PS-S NP toxicity. (**A**) Germline-specific activity of GLP-1 in controlling the transgenerational toxicity of 10 μg/L PS-S NP in inhibiting locomotion. (**B**) Neuron-specific activity of GLP-1 in controlling the transgenerational toxicity of 10 μg/L PS-S NP in inhibiting locomotion. Curves of DCL569(L4440)(PS-S NP) showed a significant difference (*p* < 0.01) compared with DCL569(L4440), and curves of TU3401(L4440)(PS-S NP) showed a significant difference (*p* < 0.01) compared with TU3401(L4440). After the PS-S NP exposure, the curves of *glp-1(RNAi)* showed a significant difference (*p* < 0.01) compared with DCL569(L4440) or TU3401(L4440).

In *C. elegans*, GLP-1 can be expressed in both the germline and the nervous system, and rare hypodermal localization was observed [66,67,68]. Further using locomotion as the endpoint, we observed that germline RNAi of *glp-1* inhibited transgenerational PS-S NP toxicity on head thrash and body bend (Figure 5A). Moreover, using TU3401 strain, neuronal RNAi of *glp-1* also suppressed transgenerational PS-S NP toxicity in locomotion (Figure 5B). Therefore, both germline GLP-1 and neuronal GLP-1 were required for inducing transgenerational PS-S NP toxicity.

### 3.8. Identification of Potential Downstream Targets of Germline GLP-1 in Controlling Transgenerational PS-S NP Toxicity

**Figure 6:**
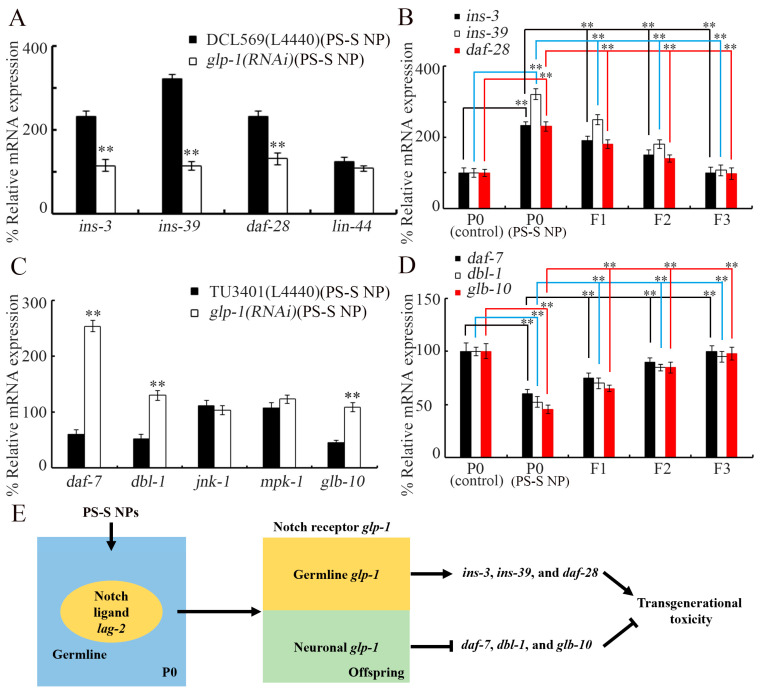
Identification of downstream targets of the germline and the neuronal GLP-1 in controlling transgenerational PS-S NP toxicity. (**A**) Effect of germline *glp-1* RNAi on expressions of *ins-3*, *ins-39*, *daf-28*, and *lin-44* in 10 μg/L PS-S NP-exposed nematodes at P0-G. ** *p* < 0.01 vs. DCL569(L4440). (**B**) Transgenerational expressions of *ins-3*, *ins-39*, and *daf-28* in 10 μg/L PS-S NP-exposed nematodes. ** *p* < 0.01. (**C**) Effect of neuronal *glp-1* RNAi on expressions of *daf-7*, *dbl-1*, *jnk-1*, *mpk-1*, and *glb-10* in 10 μg/L PS-S NP-exposed nematodes at P0-G. ** *p* < 0.01 vs. TU3401(L4440). (**D**) Transgenerational expressions of *daf-7*, *dbl-1*, and *glb-10* in 10 μg/L PS-S NP-exposed nematodes. ** *p* < 0.01. (**E**) Molecular basis for germline Notch signal in regulating transgenerational PS-S NP toxicity in nematodes.

Our previous observations have demonstrated that transgenerational PS NP toxicity can be controlled using germline insulin peptides (INS-3, INS-39, and DAF-28) and the Wnt ligand (LIN-44) in *C. elegans* [69,70]. At P0-G, germline RNAi of *glp-1* could not alter *lin-44* expression in 10 μg/L PS-S NP-exposed animals, whereas germline RNAi of *glp-1* significantly decreased the expressions of *ins-3*, *ins-39*, and *daf-28* in 10 μg/L PS-S NP exposed animals (Figure 6A). In nematodes, after the parental exposure to 10 μg/L PS-S NP, an induced increase in expressions of *ins-3*, *ins-39*, and *daf-28* could be detected from F1-G to F2-G, and recovered until F3-G (Figure 6B).

### 3.9. Identification of Potential Downstream Targets of Neuronal GLP-1 in Controlling Transgenerational PS-S NP Toxicity

In nematodes, *daf-7* and *dbl-1* encoding TGF-β ligands, *glb-10* encoding globin, *mpk-1* encoding ERK MAPK, and *jnk-1* encoding JNK MAPK act in the nervous system to control PS-NP toxicity [71,72,73,74,75]. At P0-G, neuronal RNAi of *glp-1* did not change *jnk-1* and *mpk-1* expressions in 10 μg/L PS-S NP-exposed animals, whereas expressions of *daf-7*, *dbl-1*, and *glb-10* were increased by neuronal RNAi of *glp-1* in 10 μg/L PS-S NP-exposed animals (Figure 6C). In addition, after the parental exposure to 10 μg/L PS-S NP, induced decreases in *daf-7*, *dbl-1*, and *glb-10* expressions were detected from F1-G to F2-G, and these were returned to control levels at F3-G (Figure 6D).

## 4. Discussion

Nanoplastics are an emerging environmental pollution, and widely occur in global aquatic ecosystems [76,77]. The transgenerational effects of microplastics and nanoplastics are receiving increasing attention due to risks to the health of the offspring [23]. Exposure to the pristine PS-NP induced transgenerational toxicity in both locomotion and reproduction in *C. elegans* [37,41]. However, the transgenerational effects of early-lifespan exposure to PS-S NP are still unclear. In this study, we used *C. elegans* as the animal model to assess the transgenerational toxicity of PS-S NPs. Compared with environmental mollusks and fishes, the model animal of *C. elegans* has well-described molecular and genetic backgrounds, which makes this animal model very useful for the toxicological study of pollutants. We observed that parental PS-S NP exposure caused obvious transgenerational toxicity, as verified by inhibited locomotion and impaired reproduction capability. From the mechanism of action, both parental Notch ligand *lag-2* and its receptor *glp-1* in the offspring were essential for inducing this transgenerational PS-S NP toxicity.

The nanoplastic toxicities are under the control of factors, such as size, shape, modification type, and aging degree [78,79]. The PS NPs in shorter sizes presented greater transgenerational toxicity [80]. Locomotion and reproduction are common endpoints for toxicology evaluation in *C. elegans* [34]. In nematodes, exposure to 1–100 μg/L pristine PS NP (20 nm) from L1-larvae for 6.5 days could cause transgenerational inhibition in locomotion and reproductive capacity [80]. Parental exposure to 1–100 μg/L PS-S NP (30 nm) from L1-larvae to adult day-1 could also induce transgenerational toxicity in locomotion and reproductive capacity (Figure 1B and Figure 2). In contrast, parental exposure to pristine PS NP (30 nm), at a concentration of 100 μg/L, only resulted in transgenerational toxicity in reproductive capacity [81]. This implies that exposure to PS-S NP possibly causes more severe transgenerational toxicity than pristine PS NP on organisms. In *C. elegans*, it was observed that amino-modified PS NP also exhibited more severe transgenerational toxicity in reproduction compared with pristine PS NP [81]. According to the data from published articles, the 0.1–10 μg/L were relevant to the concentrations of nanoplastics in the environment [82,83]. Our data suggested that early-lifespan exposure to PS-S NP at predicted environmental concentrations potentially causes transgenerational toxicity in organisms.

In *C. elegans*, besides epigenetic regulation signals, we assumed the existence of association between alteration in certain germline molecular signaling and transgenerational PS NP toxicity. The identification of these germline molecular signals can provide valuable bioindicators for assessing the transgenerational toxicity of nanoplastics. The Notch signaling pathway is a classical cell–cell communication, and is evolutionarily conserved in both vertebrates and invertebrates [84]. In this study, we found that the activation of germline Notch ligand LAG-2 mediated transgenerational PS-S NP toxicity. Two lines of evidence supported this. On the one hand, exposure to PS-S NP (1–100 μg/L) could only increase germline *lag-2* expression, and this increase was further detected in offspring after parental PS-S NP exposure (Figure 3A,B). On the other hand, transgenerational PS-S NP toxicity could be reversed by germline RNAi of *lag-2* (Figure 3C,D). In 1994, based on sequencing, LAG-2 was identified as the Notch ligand in *C. elegans* [85]. Previous studies have revealed the functions of LAG-2 in several aspects of *C. elegans* development, such as the patterning of precursor cell fate for the vulva and left/right asymmetry [86,87]. Our data here further demonstrated the role of LAG-2 activation in response to nanoplastics (such as PS-S NP). In previous reports, in the *C. elegans* germline, certain alterations in insulin and Wnt signals were also observed to mediate the transgenerational toxicity of nanoplastic [69,70].

Among two Notch receptors in *C. elegans*, we further found that the activation of GLP-1 was further essential for the formation of transgenerational PS-S NP toxicity. Three lines of evidence were provided to prove this. Firstly, exposure to PS-S NP (1–100 μg/L) increased *glp-1* expression, and this expressional increase in *glp-1* was also observed in offspring (Figure 4A,B). Secondly, we observed the suppression in transgenerational PS-S NP toxicity in *glp-1(RNAi)* animals (Figure 4D). In addition, in the offspring of 10 μg/L PS-S NP, *glp-1* expression was decreased by parental RNAi of *lag-2* (Figure 4C), which implied the involvement of transgenerational communication between LAG-2 and GLP-1 in mediating transgenerational PS-S NP toxicity. In *C. elegans*, GLP-1 has been confirmed as one of the corresponding receptors of Notch ligand LAG-2 [88,89]. As another receptor of LAG-2 [90], we found that the LIN-12 expression remained unchanged in nematodes exposed to 1–100 μg/L PS-S NP (Figure 4A). In nematodes, the GLP-1 is required for early embryonic cellular interaction, cell fate decisions (such as fate specification of vulva), and germline tumor formation during development [91,92,93,94]. Our results indicated that both LAG-1 activation and GLP-1 activation were involved in mediating transgenerational PS-S NP toxicity induction (Figure 6E). In *C. elegans*, the GLP-1 also participated in regulating responses to toxicants (such as quantum dots) and stresses (such as ultraviolet irradiation) [53,95].

Based on expression patterns, we performed a tissue-specific activity analysis for GLP-1 in nematodes. We found that the Notch receptor/GLP-1 functioned in germline and neurons to mediate the toxicity induction of PS-S NP in the offspring (Figure 5A,B). In nematodes, germline GLP-1 was involved in maintaining stem cells in the germline [96]. Moreover, the LAG-2 could also regulate the octanol avoidance response by activating neuronal GLP-1 [97]. Our data further confirmed the important functions of GLP-1 in these two tissues of nematodes.

The signaling from germline LAG-2 to germline GLP-1 may provide an important mechanism for forming continuous transgenerational toxicity after parental exposure to S-PS-NP. In the germline of nematodes exposed to PS-S NP, insulin peptides of INS-39, INS-3, and DAF-28 were identified as potential downstream targets of GLP-1 (Figure 6E), which further supported the role of germline GLP-1 in controlling transgenerational PS-S NP toxicity. The germline RNAi of genes encoding these three insulin peptides caused resistance to the transgenerational toxicity of PS NP [69]. During the control of transgenerational toxicity, germline insulin peptides (INS-39, INS-3, and DAF-28) in parental PS NP-exposed nematodes activated the DAF-2 in offspring, which further inhibited the activity of DAF-16 through a kinase cascade [69].

The DAF-7 and DBL-1, two TGF-β ligands, were identified as downstream targets of neuronal GLP-1 in mediating transgenerational PS-S NP toxicity (Figure 6E). Neuronal RNAi of *daf-7* and *dbl-1* caused susceptibility to PS NP toxicity [29,64]. During the regulation of nanoplastic toxicity, neuronal DAF-7 activated the activity of TGF-β receptor DAF-1 in the intestine [29], and neuronal DBL-1 activated the activity of TGF-β receptor SMA-6 in the intestine [64]. GLB-10, a globin protein, was further identified as another downstream target of neuronal GLP-1 in mediating transgenerational PS-S NP toxicity (Figure 6E). Neuronal RNAi of *glb-10* also resulted in susceptibility to PS NP toxicity [65]. During the control of PS NP toxicity, neuronal GLB-10 activated HRG-7, which further suppressed HRG-5 activity in the intestine [65].

## 5. Conclusions

Together, after the exposure of L1-larval *C. elegans* to adult day-1, 1–100 μg/L PS-S NP could cause transgenerational toxicity. Moreover, we observed that the activation of germline Notch ligand LAG-2 at P0-G mediated the transgenerational toxicity induction of PS-S NP. During the control of transgenerational PS-S NP toxicity, LAG-2 further activated corresponding Notch receptor GLP-1 in the offspring. GLP-1 acted in both germline and neurons to control PS-S NP toxicity. After exposure to PS-S NP, GLP-1 in the germline activated three insulin peptides (INS-3, INS-39, and DAF-28), and GLP-1 in the neurons suppressed DAF-7, DBL-1, and GLB-10. Our data suggested the risk of PS-S NP exposure at environmentally relevant concentrations in causing transgenerational toxicity, which was associated with the activation of germline Notch signal in organisms.

## Data Availability

Not applicable.

## Supplementary Materials

The following supporting information can be downloaded at: https://www.mdpi.com/article/10.3390/toxics11060511/s1. Figure S1: RNAi knockdown efficiency of *lag-2*. Figure S2: RNAi knockdown efficiency of *glp-1*. Table S1: Information for *C. elegans* strains. Table S2: Primer information for qRT-PCR.
