# Supplementary material for: Sulfonate-Modified Polystyrene Nanoparticle at Precited Environmental Concentrations Induces Transgenerational Toxicity Associated with Increase in Germline Notch Signal of Caenorhabditis elegans
Source: Toxics. 2023 Jun 6;11(6):511. doi: 10.3390/toxics11060511 (PMC10303405; doi:10.3390/toxics11060511)
Supplement: Supplementary file 1 [file toxics-11-00511-s001.zip › toxics-2375919-supplementary.pdf]

Supporting Information:

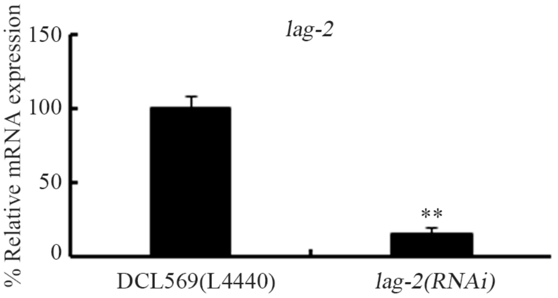

Figure S1. RNAi knockdown efficiency of *lag-2*. $^{**}P < 0.01$ *vs* DCL569(L4440).

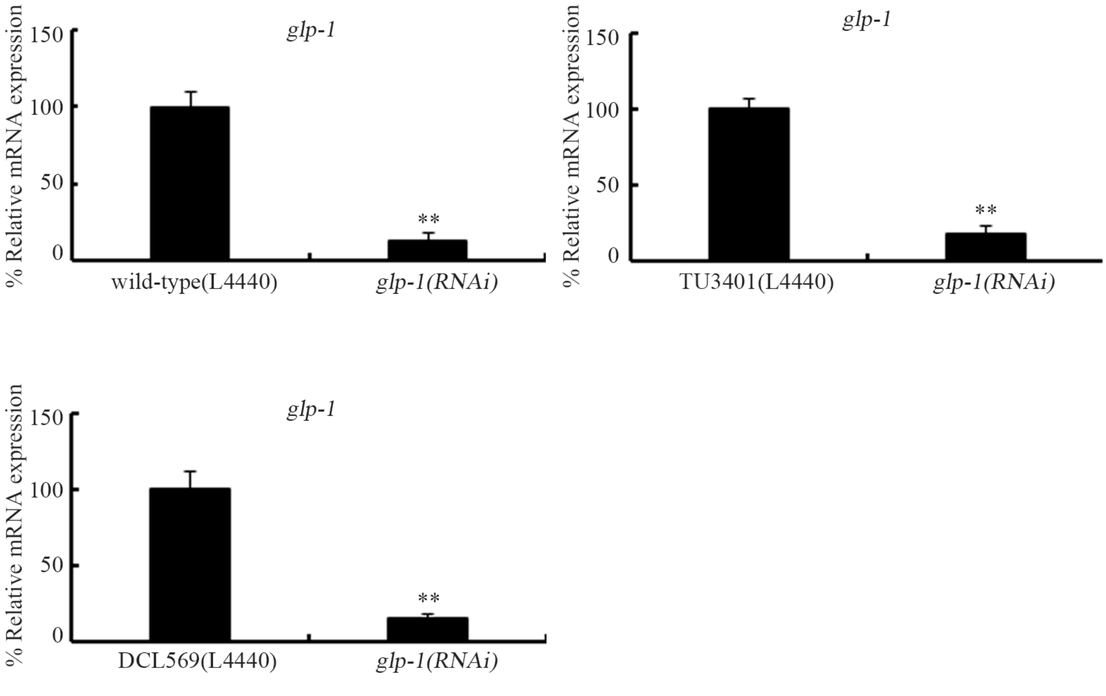

Figure S2. RNAi knockdown efficiency of *glp-1*. $^{**}P < 0.01$ *vs* wild-type(L4440), TU3401(L4440), or DCL569(L4440).

Table S1. Information for *C. elegans* strains

Strains	Genotype	Description
N2		Wild-type
TU3401	*sid-1(pk3321);[pCFJ90(myo-2p::mCherry)+unc-119p::sid-1]*	Neuronal RNAi knockdown tool
DCL569	*mkcSi13*[*sun-1p::rde-1::sun-1 3'UTR* + *unc-119*(+)]*;rde-1*(*mkc36*)	Germline RNAi knockdown tool

Table S2. Primer information for qRT-PCR

Gene	Forward primer (5'-3')	Reverse primer (5'-3')
lag-2	GACATCGGATGGATGGGACC	GGCGTCTTTGACACTGCAAG
apx-1	CGAGCTGCTCATCTCATCCC	AGCAATCCCCGTGATTTCCA
dsl-2	AGCTCGTGCTCAAAGTCGA	CCTAGGTATCGCTGAGAAGT
glp-1	TTCAACAGCGCAAAGTGTCG	TCGTTGATTCCATCGGAGGC
lin-12	TGGATCAACGTCAACACCGT	TCGGAGTATCGCGTCATTGG
ins-3	GGAGCTGCAAGTCTTATGCG	GATCTGGCCATCAGGGAGTG
ins-39	CCGAGCAAATGACTGCCAAG	CAGAGCAGGGTGACGAAGTC
daf-28	GTGGAGTGTCTGTGGAGACG	AAGAAGCAAACGTGGGCAAC
daf-7	CCCTTCATCCCCAACAGACC	GACATTGGCGATTGAGACGC
dbl-1	TGCTTCGGAAGTTGGGACTC	TTCGGTAAGGTTGTCGACGG
jnk-1	TATGCTCCACCTCCACTT	GGGTTCTTGCGTAATCTG
mpk-1	CGACTCCACGAGAAGGAT	ATATGTACGACGGGCATG
glb-10	TCAACGATCCGCGGAAAGAA	GAAATCAACATGCCGGGCTC
lin-44	ACCCTTGAGCACATTACCGA	AGCCGATCACAATCACCTTG
tba-1	TCAACACTGCCATCGCCGCC	TCCAAGCGAGACCAGGCTTCAG
